# PReS-FINAL-2296: Thrombocytopenia as a unique manifestation of antiphospholipid syndrome: a case report in the pediatric age

**DOI:** 10.1186/1546-0096-11-S2-P286

**Published:** 2013-12-05

**Authors:** LR Campos, F Sztajnbok

**Affiliations:** 1 Pediatrics, Hospital Universitario Pedro Ernesto, Universidade do Estado do Rio de Janeiro, Rio de Janerio, Brazil; 2 Pediatric Rheumatology, Adolescent Health Care Unit, Universidade do Estado do Rio de Janeiro, Rio de Janeiro, Brazil

## Introduction

The primary manifestation of the antiphospholipid antibody syndrome (APS) is thrombosis, which forms the core of the classification criteria for this syndrome. However, multiple other noncriteria manifestations have been attributed to APS, some of which do not appear to have thrombosis as part of the pathophysiology, although they may be mediated by autoantibodies. Examples of these manifestations include neuropathy, thrombocytopenia, and cardiac valvular disease. Of importance, these manifestations of APS may not respond well to anticoagulation, and therefore additional therapies are needed.

## Objectives

To describe a case report of a 12-years old boy with severe isolated thrombocytopenia that was diagnosed as antiphospholipid syndrome after an extended diagnostic work-up.

## Methods

Case report and review of the literature.

## Results

We had a case of a 12-years old boy with severe isolated thrombocytopenia (as low as 20.000/mm^3^) that was investigated for hematological diseases and previously treated for a year for chronic immune thrombocytopenic purpura without resolution with steroids. After an evaluation by a rheumatologist, persistent high titers of IgM anticardiolipin, IgM anti-β2 glycoprotein, and positive lupus anticoagulant were found.

A diagnosis of thrombocytopenia as an isolated manifestation related to antiphospholipid-syndrome was made. There were no other remarkable symptoms, clinical findings, laboratory tests or family history.

He presented a good initial response to corticosteroids, but platelets decreased rapidly with dose reduction. He did not show a good response to hydroxychloroquine. Treatment with IVIg was started with very good response, but platelet rapidly dropped to very low levels three weeks after each infusion and no changes in antibody titers were noted after six infusions. Rituximab (anti-CD20 monoclonal antibody) was started and platelets increased rapidly a month after infusion. After 10-months of a single dose of rituximab, platelets are still high (300.000/mm^3^), anticardiolipin IgM and anti-β2 glycoprotein IgM titers are lower than before. Immunoglobulin levels are still in the normal range and CD19 and CD20 are low. Currently, he is on hydroxychloroquine and aspirin.

## Conclusion

Despite causing no substantial change in aPL profiles, rituximab may be effective in controlling thrombocytopenia in antiphospholipid syndrome.

## Disclosure of interest

None declared.

